# Special Issue: Yeast Cell Signaling Pathways (Volume 1)

**DOI:** 10.3390/ijms24054929

**Published:** 2023-03-03

**Authors:** Vitor Teixeira

**Affiliations:** 1 i3S-Instituto de Investigação e Inovação em Saúde, Universidade do Porto, 4200-135 Porto, Portugal; vitor.teixeira@ibmc.up.pt; 2 IBMC-Instituto de Biologia Molecular e Celular, Universidade do Porto, 4200-135 Porto, Portugal

This Special Issue was devoted to unravelling novel aspects of yeast biology and signal transduction in numerous yet intricate basic processes. The work presented herein sheds additional insights into our current understanding of living organisms at a cellular level, using yeast as a privileged platform for investigation and systems biology.

The yeast cell wall integrity pathway (CWI) pathway has evolved in fungi as part of the adaptive stress mechanisms in response to environmental stimuli. Its activation leads to an overall transcriptional reprogramming involving genes related to cell wall biogenesis and remodeling, morphogenesis, bud formation, and hence cell division. As part of one of the mitogen-activated protein kinase (MAPK) pathways, it comprises a three-tiered protein kinase cascade composed of the MAKKK Bck1, the MAPKKs Mkk1 and Mkk2, and the MAPK Slt2 s [1]. Using a previously developed platform so-called Integrity Pathway Activation Circuit (IPAC), which is based on a rewired genetic circuit leading to hyperactivation of the CWI pathway [2], Jiménez-Gutiérrez, Fernández-Acero et al. employed this technology to screen novel compounds capable of activating the CWI cascade [3]. They found that neomycin, contrarily to other the aminoglycosides, activates the CWI pathway, and they were able to define the corresponding components, namely the mechanosensor Wsc1, the Rho1 Guanine nucleotide exchange factor (GEF) Rom2, the MAPKKK Bck1, the MAPKK Slt2, and the SCB binding factor (SBF) transcription factor components Swi4 and Swi6. Based on the rationale that neomycin is able to sequester phosphatidylinositol 4,5-bisphosphate [PtdIns (4, 5) P2], Jiménez-Gutiérrez, Fernández-Acero and colleagues were enthralled with the possibility that neomycin might cause plasma membrane stress by changing PtdIns (4, 5) P2 dynamics. They eventually showed that neomycin reduces the availability of this lipid at the plasma membrane, consistent with changes observed in the distribution of a PtdIns (4, 5) P2 fluorescent reporter. In addition, they revealed that neomycin interferes with the TORC2 pathway, which is known to be triggered in response to changes in plasma membrane tension promoted by osmotic shock or inhibition of sphingolipid biosynthesis [4]. However, it would be interesting to evaluate how neomycin affects TORC2 activity. This could potentially involve changes in PtdIns (4, 5) P2 phase separation and plasma membrane tension, consistent with the observed PtdIns (4, 5) P2 spots in the cell periphery of neomycin-treated cells, and alterations in Slm1/2 protein recruitment to TORC2-containing domains.

Sphingolipids belong to a cohort of bioactive lipids with dissectible functions in numerous aspects of cellular metabolism and physiology, including nutrient availability, endocytosis, autophagy, senescence, apoptosis and cell growth [5]. Sphingolipids are closely related to the activation of signaling pathways that influence both cell size and growth rate, namely TORC2 [6] and Sch9 [7]. In this issue, Flor-Parra et al. have shown that ceramide synthase Lac1 (but not Lag1) influences cell growth and size in the fission yeast *Schizosaccharomyces pombe* [8]. The authors propose that such differences might be related to different profiling in terms of ceramide species and complex sphingolipids, thus accounting for the specific defects in cell growth and size found for *lac1*∆ cells. In this regard and as previously proposed, Lag1 and Lac1 are not redundant enzymes and seem to fulfill distinct roles depending on the cellular context [9].

Autophagy is a highly conserved basic cellular process that plays critical roles under physiological and pathophysiological conditions, ranging from degradation of cytosolic components, recycling of nutrients, protection against metabolic stress and DNA damage, maintenance of proteostasis and defense against intracellular pathogens [10]. Numerous studies have drawn multiple correlations between autophagy dysfunction with the progress of disease in association with apoptosis and cell death, activation of stress response pathways and alterations in redox homeostasis [10]. Although we have already grasped key features of this stepwise process in terms of the protein complexes and their regulation, the definition of the mechanisms that cells employ to spatiotemporally regulate autophagy at different stages remains rather elusive. To define the signaling events related to phosphatidylinositol 3-phosphate [PI(3)P] generation and protein complex assembly, which is essential for autophagy initiation and progression, Zhao, You et al. decided to investigate if Rab5/Vps21 regulates PI3K complex and PI(3)P localization to the phagophore assembly site (PAS) and how this is spatially and temporally related to the recruitment of Atg5–Atg12·Atg16 and Atg8-PE (LC3-II) to the PAS [11]. In this study, they showed that the membrane-binding component of the CORVET complex Vps8 (but not Vps3) interacted with Vps34 and Atg21 in a Vps21-dependent manner on endosomes. However, it remains unclear how PI(3)P generation and PI(3)P-binding proteins location to these domains are regulated by Vps21 during this process. Importantly, Vps21 regulates the recruitment of the two conjugation systems, Atg5-Atg12·Atg16 and Atg8-PE (LC3-II), by targeting Atg21, which interacts with Atg16 or Atg8. The Vps21 homolog RAB5 was found to regulate early steps of autophagosome formation by controlling the recruitment of the autophagic BECN1-PIK3C3 complex and subsequent PI(3)P production on the phagophore membrane. It also adjusts the conjugation of ATG12 to ATG5 through its effector, PIK3C3, as member of the autophagic BECN1 complex [12]. Whether it acts through Atg21 in mammalian systems remains to be determined.

Apart from autophagy, other intracellular trafficking pathways are important for targeting and the turnover of plasma membrane proteins, which include endosomal recycling routes. In recent years, efficient recycling of cargoes from endosomes have uncovered that several post-translation modifications (PTM) of proteins regulate their trafficking. Importantly, protein acetylation, which refers to the covalent binding of an acetyl group to the N-terminus or a lysine residue of a protein, has been recently implicated in such processes. This PTM is mediated by acetylases, such as lysine acetyltransferase (KAT) enzymes, and reversed by deacetylases, including various lysine deacetylases (KDAC) [13]. Much has been learnt about the acetyltransferase and deacetylase enzymes that regulate protein-DNA and protein–protein interactions. How it is involved in endosomal trafficking and membrane remodeling is still largely unknown. Using an array of microscopy, biochemical and bioinformatics approaches, Amoiradaki, Bunting et al. performed a systematic interrogation of haploid deletion mutants defective in endosomal recycling by employing fluorescent-based probes [14]. They identified the yeast Rpd3, functionally related to the mammalian KDAC Rpd3/Hda1 family, and other subunits of the Rpd3 complex. However, not all members of the Rpd3 complex are required for efficient transcriptional control of the recycling pathway, and many of them are necessary to different degrees depending on specific contexts used to screen such factors. Using a suppressor strategy of *rpd3*Δ recycling defects based on gene overexpression, they also identified the protein phosphatase Sit4, the transcriptional regulator Ldb7 and the sporulation factor Dit1. They also showed that Pik1 could play a role in recycling, most likely by membrane lipid remodeling necessary for Golgi and endosomal trafficking via the Rpd3 complex. Indeed, acetylation can have diverse effects on peripheral membrane proteins and their functions. The ability of acetylation to change protein–membrane interactions and alter the specificity of lipid binding can affect a wide range of cellular processes, including membrane trafficking, signaling, and protein activity. For instance, membrane proteins containing BAR, PX, C2, or EHD membrane-binding domains undergo lysine acetylation in *Drosophila* and they have been implicated in membrane trafficking and remodeling events [15]. In addition, in proteins interacting with highly negatively charged lipids (e.g., phosphoinositides), the loss of positive charge via acetylation may shift the specificity to a less acidic or even neutral lipid. One can envision that this would affect membrane localization and/or enzyme activity. Therefore, understanding the mechanisms and consequences of protein acetylation is essential for comprehensively elucidating the complex signaling pathways that regulate membrane-related cellular events.

Lipid droplets (LDs) are ubiquitous metabolic hubs made of triacylglycerol (TAG) and sterol esters (SEs), which are encased by a phospholipid monolayer coated with various proteins prominently dedicated to lipid dynamics. Important, LDs fulfill crucial roles in many aspects of cellular physiology and metabolism, including lipid metabolism, autophagy, stress response and cell fate [16]. As lipid depots and signaling platforms, the processes of lipid storage and mobilization are very well regulated according to the type of tissue and the developmental and metabolic stage of the cell. However, our current understating of the signaling pathway governing LD dynamics is still very limited. In that regard, Teixeira and colleagues [17] have recently advanced the field by showing that major regulators of cellular growth, namely Target of Rapamycin Complex 1 (TORC1), Protein kinase A (PKA) and the vacuolar ATPase (V-ATPase) are important for cellular neutral lipid accumulation under specific conditions. Importantly, it was shown that the expression of one of the TAG-synthesizing enzymes DGA1/DGAT was controlled by the PKA/TORC1 regulated transcription factor Sfp1. Using different approaches, they provided strong evidence that cytosolic pH controls membrane biogenesis by modulating the localization and activity of the transcription repressor Opi1. Opi1 is an ER-resident protein that senses alterations in cellular PA concentration, allowing feedback regulation of lipid metabolism. Moreover, Opi1 binds phosphatidic acid (PA) via its Q2 domain, which is pH sensitive. In this work, they provided evidence that either the loss of V-ATPase or the plasma membrane H^+^-ATPase Pma1 activities leads to LD accumulation. Mechanistically, it was demonstrated that cytosolic acidification due to inability to maintain intracellular pH homeostasis decreases Opi1 affinity toward PA and affects its location to potentiate its transcriptional repressor activity, thus favoring lipid storage over membrane proliferation. Overall, this work provided new insights in the molecular interplay between the activity of major signaling effectors and pH homeostasis during membrane growth and lipid storage.

Although Sfp1 is most notably known for its role in the transcriptional control of ribosomal protein (RP) genes in response to various physiological and environmental cues [18,19], its role in stress response [20] has been expanded to infection and innate immunity with the work of Hsu, Liao, Chang et al. [21]. They provided evidence that the human antimicrobial peptide (AMP) LL-37 triggers cell wall remodeling and the Endoplasmic Reticulum (ER) stress response, which in turn promote oxidative damage, possibly related to changes observed in Ero1-mediated oxidative protein folding, and alterations in protein secretion during *Candida albicans* fungal infection. Remarkably, the cell response to LL-37 is Sfp1-regulated since they showed that Sfp1 deficiency significantly ameliorated these phenotypes and increased survival of the fungus to LL-37. Whether Sfp1 coordinates these various signaling cascades in response to LL-37 and possibly other AMPs is unclear at this stage.

The capability of any cell to adhere to and interact with surfaces is a defining feature for the survival in evolving and multifaceted environments. Hence, organisms have developed distinct strategies based on specific and/or non-specific interactions for adherence and attachment to a specific surface. Whereas adhesion to abiotic surfaces is mostly dictated by non-specific and transient contacts, adhesion to biotic surfaces typically requires a specific receptor-ligand interaction and for that surface adhesion proteins (adhesins) are essential for cellular adhesion to surfaces. In both cases, these interactions share the same fundamental physicochemical forces, and mechanical processes are involved in nearly every facet of surface adhesion. To highlight it, Lipke et al. [22] provided the reader with a comprehensive overview of cell adhesion processes, mating adhesins, as well as the corresponding specificities and affinities in terms of interaction, in several eukaryotic organisms. Importantly, it examines the potential and the progress already brought by single-cell force spectroscopy (SCFS) methodologies, namely atomic force microscopy (AFM), which are well suited to define the nature of single-molecular interactions in terms of the mechanical properties and biophysical dynamics behind such adhesive interfaces. With multiple applications not limited to eukaryotes phenomena, it will ultimately improve our understanding of cell–substrate adhesion, cell–cell adhesion, and cell–host adhesion processes at the molecular level.

The metabolic engineering goal of extending substrate range in microorganisms used in biotechnological processes has become the *holy grail* for this industry. The engineering of baker’s yeast *Saccharomyces cerevisiae* to utilize D-xylose, a five-carbon sugar, has become a prominent example. While many exceptional engineering strategies have been developed using the budding yeast to ferment D-xylose to ethanol at high yields, the consumption rate of D-xylose is still significantly lower than that of its preferred sugar D-glucose, which represents a limiting step in its utilization for biotechnological purposes. To address this, the response of *S. cerevisiae* signaling pathways to D-xylose has emerged as a promising and prominent engineering target. In a comprehensive review, Brink et al. first compiled the response of the sensing and signaling pathways to D-glucose, and then discussed the known signaling response to D-xylose in *S. cerevisiae* and current attempts to improve the response by signaling engineering using both native targets and synthetic (non-native) regulatory circuits [23]. They also discussed the challenges involved in achieving economically feasible yields, titers, and productivities in bioprocesses, particularly those involving engineered microbial cell factories with expanded substrate ranges. They noted that D-xylose sensing by engineered yeast has good potential to become a standard platform in the field of non-natural substrate sensing and signaling, due to the substantial metabolic engineering achievements already in place and the well-studied topic of native D-glucose sensing in yeast. However, it also acknowledges that a lot remains to be understood about the signaling responses to different sugars before rational signaling engineering can be attempted at a larger scale. Overall, this work highlights the importance of molecular optimization and engineering of substrate sensing and signaling for achieving industrially and societally relevant bioprocesses using microbial cell factories.

In the past years, research in the field of cell division and cancer biology has reported an important role of the conserved E3-ubiquitin ligase complex, Anaphase-Promoting Complex/Cyclosome (APC/C), in these processes [24]. Apc1 and Apc5 are two subunits of the APC/C, a large multi-subunit protein complex that plays a critical role in cell cycle progression by targeting specific proteins for degradation during mitosis. The APC/C requires the co-factors Ccs52 and Cdc20 to activate its ubiquitin ligase activity and target specific substrates for degradation [24]. There is growing interest in targeting the interaction between Cdc20 and the APC/C in cancer therapy, as dysregulation of this interaction has been implicated in several types of cancer. In particular, recent studies have suggested that targeting a specific N-terminal region of Cdc20 that interacts with Apc1 and/or Apc5 may be an effective strategy for disrupting this interaction and inhibiting the growth of cancer cells. One challenge in targeting this interaction is the development of drugs that specifically block the interaction between Cdc20 and Apc1 and/or Apc5 without affecting other cellular processes. However, the detailed knowledge of the structure and function of the APC/C in yeast [25] provides a strong foundation for the identification and characterization of potential drug targets, and the use of budding yeast as a model system may be an effective tool for developing and testing potential drug candidates. In their review, Schuyler and Chen stated that despite potential challenges and uncertainties, the deep understanding of cell cycle signaling pathways in yeast still can be leveraged to identify small molecules that enhance the ability of mitotic poisons to disrupt cancer cell progression and induce cell death [26]. The wealth of molecular and cellular tools available in budding yeast makes it a strong model system for identifying and characterizing specific molecular targets for drug discovery, such as potential Apc1-Cdc20 and/or Apc5-Cdc20 interaction sites. The hope is that this knowledge can be employed to identify molecules that specifically inhibit APC/C-Cdc20, causing an extended mitotic arrest in the presence of a mitotic poison without affecting the function of the mitotic checkpoint complex.

## Data Availability

Not applicable.
